# Supplementary material for: Addressing bias in national population density models: Focusing on rural Senegal
Source: PLoS One. 2024 Nov 12;19(11):e0310809. doi: 10.1371/journal.pone.0310809 (PMC11556701; doi:10.1371/journal.pone.0310809)
Supplement: S1 Table — (DOCX) [file pone.0310809.s001.docx]

## Result of random forest hyperparameter tuning

S1 Table: Results of hyperparameter tuning

| High population model | | | | Middle high population | | | | Middle low population | | | | Low population | | | |
| --- | --- | --- | --- | --- | --- | --- | --- | --- | --- | --- | --- | --- | --- | --- | --- |
| RMSE | mtry | node_size | sample_size | RMSE | mtry | node_size | sample_size | RMSE | mtry | node_size | sample_size | RMSE | mtry | node_size | sample_size |
| 0,76 | 16 | 7 | 367 | 0,35 | 4 | 3 | 198 | 0,29 | 2 | 5 | 188 | 0,28 | 2 | 8 | 78 |
| 0,80 | 14 | 6 | 336 | 0,31 | 7 | 2 | 261 | 0,29 | 2 | 10 | 169 | 0,28 | 4 | 4 | 89 |
| 0,86 | 7 | 3 | 381 | 0,31 | 9 | 2 | 255 | 0,31 | 6 | 2 | 156 | 0,20 | 7 | 3 | 86 |
| 0,36 | 3 | 1 | 241 | 0,31 | 3 | 8 | 277 | 0,26 | 5 | 2 | 173 | 0,29 | 2 | 6 | 87 |
| 0,75 | 8 | 7 | 383 | 0,34 | 8 | 4 | 285 | 0,33 | 3 | 6 | 162 | 0,25 | 3 | 8 | 80 |
| 0,35 | 12 | 1 | 335 | 0,30 | 10 | 1 | 277 | 0,32 | 3 | 1 | 156 | 0,28 | 2 | 1 | 78 |
| 0,99 | 24 | 7 | 306 | 0,34 | 7 | 2 | 198 | 0,25 | 5 | 3 | 173 | 0,29 | 3 | 5 | 87 |
| 0,73 | 17 | 1 | 394 | 0,31 | 3 | 6 | 261 | 0,33 | 3 | 2 | 162 | 0,21 | 6 | 1 | 86 |
| 0,72 | 22 | 1 | 366 | 0,32 | 7 | 6 | 255 | 0,32 | 2 | 3 | 169 | 0,27 | 1 | 9 | 80 |
| 0,80 | 7 | 2 | 307 | 0,33 | 4 | 8 | 285 | 0,29 | 2 | 4 | 188 | 0,32 | 2 | 6 | 89 |
| 0,37 | 7 | 2 | 294 | 0,32 | 2 | 3 | 255 | 0,26 | 5 | 1 | 173 | 0,28 | 5 | 5 | 94 |
| 0,78 | 10 | 2 | 352 | 0,31 | 11 | 4 | 277 | 0,29 | 1 | 9 | 169 | 0,30 | 1 | 10 | 92 |
| 0,77 | 15 | 4 | 378 | 0,34 | 5 | 5 | 198 | 0,29 | 24 | 2 | 156 | 0,29 | 15 | 3 | 84 |
| 0,76 | 14 | 5 | 363 | 0,33 | 11 | 6 | 285 | 0,29 | 2 | 4 | 188 | 0,18 | 7 | 1 | 74 |
| 0,95 | 36 | 10 | 321 | 0,32 | 5 | 1 | 261 | 0,31 | 20 | 1 | 162 | 0,26 | 9 | 1 | 76 |
| 0,83 | 8 | 9 | 307 | 0,31 | 2 | 4 | 261 | 0,32 | 18 | 2 | 153 | 0,28 | 3 | 2 | 89 |
| 0,78 | 19 | 10 | 366 | 0,30 | 4 | 4 | 277 | 0,33 | 3 | 1 | 150 | 0,20 | 2 | 1 | 86 |
| 0,79 | 9 | 1 | 394 | 0,34 | 4 | 5 | 285 | 0,32 | 2 | 3 | 187 | 0,28 | 6 | 4 | 87 |
| 1,00 | 18 | 1 | 306 | 0,35 | 1 | 3 | 198 | 0,26 | 2 | 1 | 182 | 0,27 | 2 | 9 | 80 |
| 0,35 | 10 | 1 | 335 | 0,31 | 1 | 8 | 255 | 0,28 | 5 | 8 | 176 | 0,29 | 1 | 4 | 78 |
| 0,37 | 7 | 4 | 294 | 0,31 | 5 | 4 | 261 | 0,29 | 2 | 7 | 169 | 0,29 | 4 | 1 | 78 |
| 0,98 | 13 | 7 | 321 | 0,34 | 5 | 5 | 285 | 0,26 | 14 | 2 | 173 | 0,27 | 1 | 6 | 80 |
| 0,77 | 15 | 1 | 378 | 0,31 | 7 | 5 | 277 | 0,29 | 3 | 5 | 156 | 0,28 | 2 | 3 | 89 |
| 0,78 | 8 | 2 | 363 | 0,31 | 6 | 1 | 255 | 0,31 | 2 | 4 | 162 | 0,29 | 1 | 5 | 87 |
| 0,76 | 13 | 1 | 352 | 0,38 | 8 | 2 | 198 | 0,31 | 5 | 2 | 188 | 0,20 | 7 | 5 | 86 |
