# Supplementary figures and images for: Addressing bias in national population density models: Focusing on rural Senegal
Source: PLoS One. 2024 Nov 12;19(11):e0310809. doi: 10.1371/journal.pone.0310809 (PMC11556701; doi:10.1371/journal.pone.0310809)

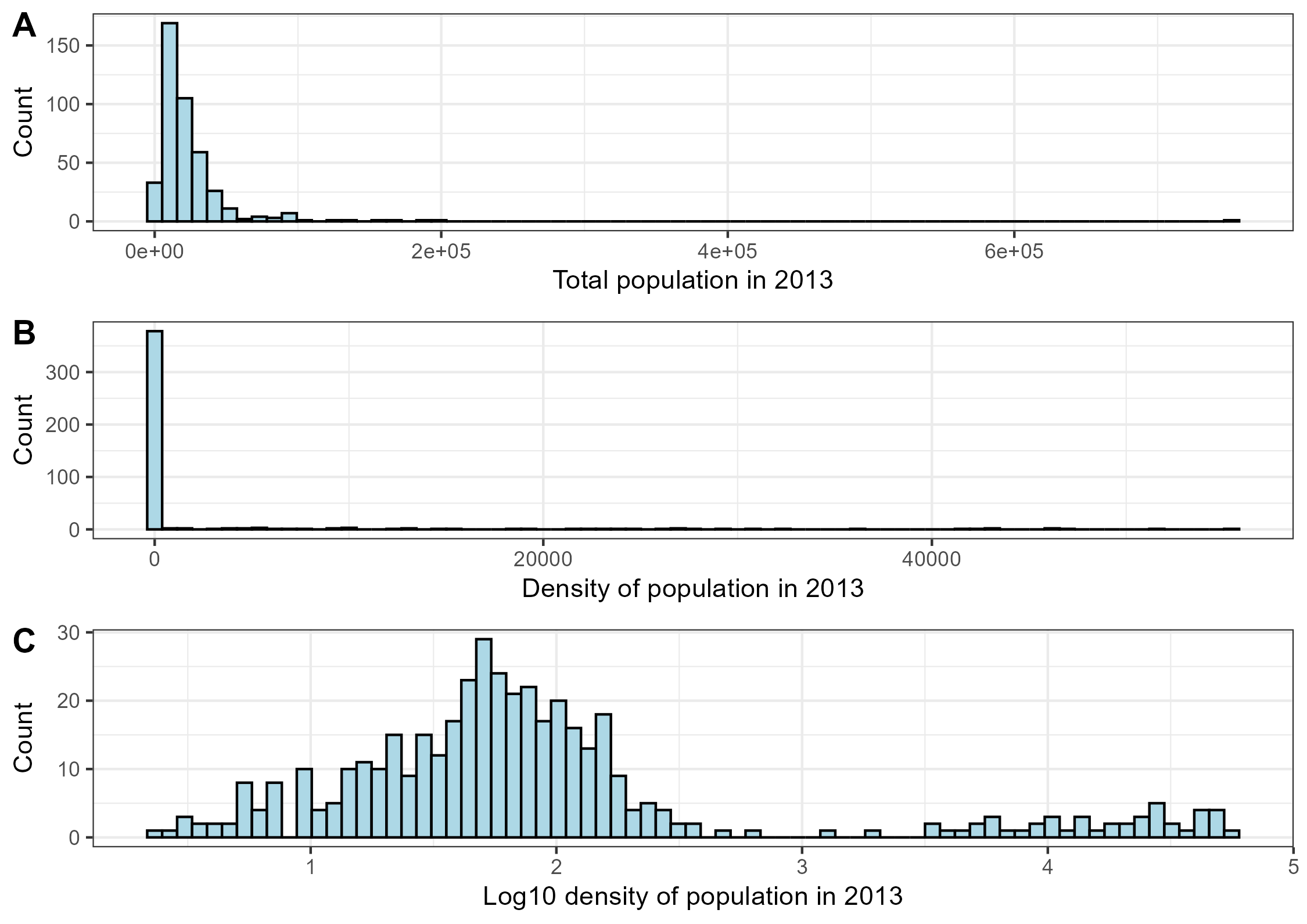

Supplement: S1 Fig — Population metrics for Senegal in 2013, (A) is total population count per administrative unit, (B) is population density per administrative unit (#hab/km2) and (C) is the log density of population per administrative unit. (TIF) [file pone.0310809.s002.tif]
